# “A fog that impacts everything”: a qualitative study of health-related quality of life in people living with HIV who have cognitive impairment

**DOI:** 10.1007/s11136-022-03150-x

**Published:** 2022-05-17

**Authors:** Kate Alford, Stephanie Daley, Sube Banerjee, Elizabeth Hamlyn, Daniel Trotman, Jaime H. Vera

**Affiliations:** 1 Department of Global Health and Infection, Brighton and Sussex Medical School, Brighton, UK; 2 Centre for Dementia Studies, Brighton and Sussex Medical School, Brighton, UK; 3 Faculty of Health, University of Plymouth, Plymouth, UK; 4 King’s College Hospital NHS Foundation Trust, London, UK; 5 University Hospitals Sussex, Sussex, UK

**Keywords:** HIV, Cognitive impairment, Quality of life, Qualitative, HIV-associated neurocognitive disorder

## Abstract

**Background:**

Cognitive impairment (CI) in people living with HIV (PLWH) is an important health concern in the context of an ageing HIV population. Impacting 14–28% of PLWH, CI is associated with lower health-related quality of life (HRQoL), however, evaluation of the illness-specific factors comprising HRQoL in PLWH with CI have not been assessed.

**Objective:**

We sought to contribute evidence toward an understanding of HRQoL and identify domains of HRQoL in PLWH with CI.

**Methods:**

Qualitative interviews with 25 PLWH with objective CI related to HIV disease were conducted with participants attending HIV clinics in the UK. Clinically significant CI was defined based on The European AIDS Clinical Society guidelines, requiring: (i) subjective reporting of cognitive symptoms; (ii) symptoms to be related to HIV (e.g. potentially confounding non-HIV related conditions have been excluded or are being optimally managed) and; (iii) formal neuropsychological assessment confirming CI. Median age was 56 years (range 35–80); 18 participants were men (72%); 11 (44%) were white British and 8 (32%) were Black African; 14 (56%) were men that have sex with men and 10 (40%) were heterosexual; median number of years living with HIV was 17 (range 1–34); and all participants were on combination antiretroviral therapy. Analyses employed techniques from grounded theory, underpinned by an inductive, collaborative team-based approach.

**Results:**

Findings revealed seven interrelated domains comprising HRQoL experiences were identified: Physical function, Cognition, Social connectedness, Physical and mental health, Stigma, Self-concept, and Control and acceptance, and each was defined by specific descriptive components.

**Conclusion:**

This study provides valuable insights on the factors that drive HRQoL in PLWH with CI and contribute to a body of evidence which provides targets for the development of targeted interventions to maintain or improve quality of life.

## Plain English summary

Cognitive impairment related to HIV disease is being seen more in people living with HIV. Particularly in older adults living with HIV. This is likely due to a combination of long-term medication use, damage caused to the brain by the HIV infection, and natural ageing. Studies suggest up to 28% of people living with HIV have cognitive difficulties. However, in healthcare focus tends to be on diagnosing and understanding the cause of the cognitive impairment and not on helping with the wider effects of living with both HIV and cognitive difficulties. Other research finds people living with HIV and cognitive impairment report particularly poor quality of life. These individuals are often social isolated, unemployed, and experience mental health disorders. We conducted a series of in-depth interviews to explore health-related quality of life in this group. We identify seven main areas which appear important to quality of life. These were physical functioning, cognition, social connectedness, physical and mental health and wellbeing, stigma, self-concept, and control and acceptance. This study provides insights into the factors that affect quality of life in those with HIV and cognitive impairment. Understanding this provides targets for interventions which may have a meaningful benefit to the lives of those living with this condition.

## Introduction

Cognitive impairment (CI) due to HIV disease affects 14–28% of people living with HIV (PLWH) [1], and impacts on medication adherence, employment, and quality of life [2]. Recent cohort studies have estimated 73% of Europe’s estimated 2.2 million [3] PLWH will be over 50 years of age by 2030 [4]. With the attenuation of cognitive reserve seen in PLWH, as well as an increased incidence of neurodegenerative disorders with age, there is likely to be an increased burden of cognitive difficulties in PLWH in coming years.

The advent and availability of effective antiretroviral medication in the last 20 years has transformed HIV from a life-limiting illness to a chronic condition which can be successfully managed on a long-term basis. In the UK, men who have sex with men (MSM) and Black African people are disproportionally affected [5] and research generally reports health-related quality of life (HRQoL) in PLWH as being worse than that seen in non-infected populations [6–9]. PLWH have to cope with a range of HIV-related symptoms and experience more co-morbidities (including cognitive impairment), than age-matched negative populations [10, 11]. In addition to the biological consequences, PLWH frequently face wide-ranging psychosocial influences such as stigma, poverty, substance abuse, poor mental wellbeing, and cultural beliefs which can impact HRQoL [12]. HRQoL is a multi-dimensional construct comprised of aspects including physical, psychological, and social functioning. Generic models (i.e. those applying to all illnesses) may also include domains of general health perceptions, along with dimensions of pain, energy, independence, environment and spirituality [13–18]. Generic models may miss components of HRQoL that are important in particular disorders, so disease-specific conceptualisations and measures have been developed to identify and measure domains of HRQoL which have particular salience in specific illness experiences. For PLWH, alongside generic aspects, domains of stigma and discrimination, barriers to care, engagement coping, cognition, and sexual functioning have been identified as important [19–22]; in individuals with mild/moderate dementia domains of self-concept, behaviour and mood, and self-awareness have been identified as central to HRQoL [23–26]. HRQoL provides a broad and patient-reported appraisal of the impact of a condition on that individual and the overall effects of treatment and care, and so are of increasing importance in the holistic evaluation of interventions and services. Therefore, understanding more about HRQoL in PLWH with CI might assist in the provision of improved person-centred care and inform the development of targeted interventions to maintain or improve quality of life.

The clinical characteristics of CI in the context of HIV (often referred to as HIV-associated Neurocognitive Disorder (HAND), in research contexts [27]) are well documented with biomedical and neuropsychological studies reporting deficits in memory, learning, attentional and executive processes which have impact on health and function [28]. Less is known about the impact of these impairments with a recent scoping review of research finding a paucity of studies examining HRQoL and its associated dimensions in PLWH with CI [29]. Quantitative studies report lower overall HRQoL, and attenuated physical, psychological, and social HRQoL in PLWH with CI, compared to HIV [30–32] and non-HIV matched controls without CI [32]. Specifically, these studies found PLWH with CI reported poorer overall QoL across all domains of the MOS-HIV questionnaire [30, 31] and the SF-36 [32] which include domains related to physical functioning, physical health, mental health, and social functioning. Three qualitative studies, have sought to understand the lived experiences of PLWH with CI, and found impacts on concentration, multi-tasking, and fulfilling daily activities) [2, 33, 34], negative emotional reactions (embarrassment, stress, worry, depressive symptoms) [2, 33], and dimensions of discrimination and stigma [2] that affect them in work and social settings. These studies, along with the calls to include good quality of life as a fourth ‘90’ in the UNAIDS Testing and Treatment targets, support the value of further work to assess HRQoL directly and the illness-specific domains comprising this important outcome in PLWH with CI. Here we describe the results of a study conducted systematically to understand HRQoL experiences and identify domains of HRQoL in PLWH with CI.

## Methods

### Study design

We conducted an exploratory qualitative study with participants recruited from specialist HIV clinics in the South-East of England from January to July 2020. Analysis of transcripts and decisions regarding the final domains were led by an experienced qualitative researcher (SD) and the team included a doctoral student (KA), an HIV medical consultant/academic (JV), and a Dementia medical consultant/Professor of Dementia (SB).

### Participants

Participants were patients attending specialist HIV services in London (King’s College Hospital) or Brighton (University Hospitals Sussex). All had a diagnosis of HIV, were on combination antiretroviral therapy, and had clinically significant CI. Clinically significant CI was defined based on the European AIDS Clinical Society (EACS) [35] definition of CI related to HIV. This requires: the patient reporting subjective symptoms of CI; the HIV physician considering the symptoms to be related (partly or completely) to HIV disease (e.g., potentially confounding non-HIV related conditions have been excluded or are optimally managed); and formal objective neuropsychological assessment, based on multiple cognitive domain testing, confirming CI. Patients who were judged by their HIV consultant to be unable to give informed consent were not considered eligible.

### Procedure

Clinicians in each HIV service advised potential participants about the study and referred interested participants to researchers. All participants gave informed consent prior to participating. Interviews were conducted face-to-face (*n* = 10) or over the telephone (*n* = 15) depending on patient preference. Interviews lasted 45–97 min and were audio-recorded, transcribed verbatim and checked for accuracy. Interview guides based on a review of the HRQoL literature and experiences of PLWH with CI [29] were used to structure the interview. Questions were open-ended, and probing. Key issues explored included: experiences of living with HIV and CI; how the conditions impact on one another; personal understanding of quality of life; and effect of conditions on HRQoL and on associated domains (impact of difficulties on function, physical health, psychological health, social experiences, and relationships). See Table 1 for the interview key questions and probes. Severity of CI was determined based on most recent MoCA (Montreal Cognitive Assessment [36]) score, which is widely used and well validated screening instrument used in HIV clinics.

**Table 1:** Interview guide—key questions and probes

(1) What is your experience of living with HIV?
(How does it affect your life, in what ways)
(2) What is your experience of living with cognitive or memory issues?
(How does it affect your life, in what ways, when did you notice it, what was your experience of diagnosis)
(3) How is it living with both HIV and CI? How do they affect one another? Is one more impactful than the other?
(4) What is your understanding of what quality of life is? What does it mean to you?
(5) How is your quality of life affected by living with HIV and having cognitive problems?
(How does it affect the things you enjoy, your ability to do chores/housework/things you need to do, your family life/relationships/friendships, how you feel about yourself, your mental health—in what ways?)
(6) How do you manage or cope with these issues?
(Compensatory or remediation strategies, spirituality, support networks, healthcare access/support?)
(7) Other than HIV or cognitive issues what else affects your quality of life?
(8) What are the main things which negatively affect your quality of life?

### Data analysis

**Fig. 1:**
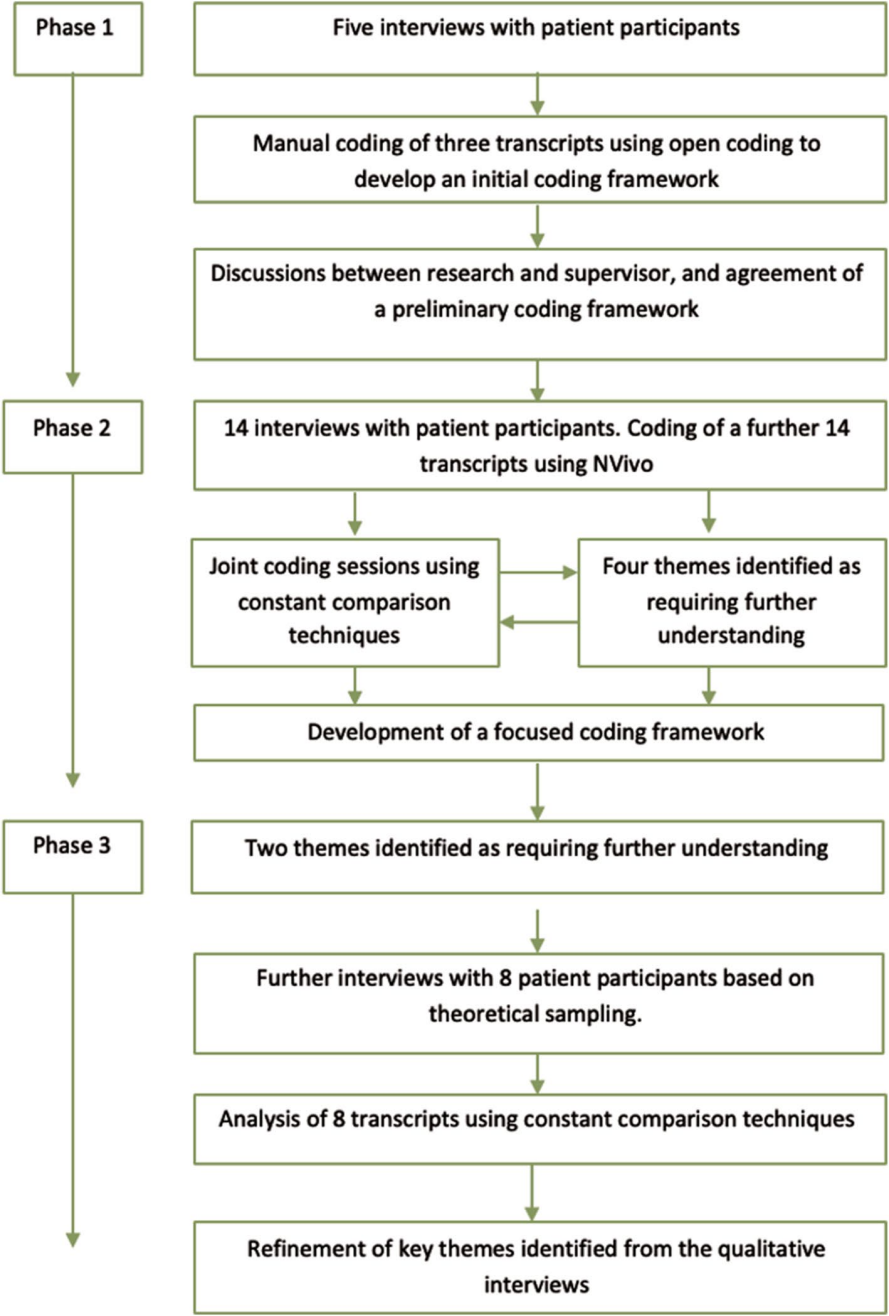
Process of data collection and analysis

A team-based analysis of interview transcripts was conducted to identify components and domains relating to the impact of CI on the HRQoL of PLWH. Data were analyzed using techniques from grounded theory, informed by Hardy and Bryman’s (2004) adapted framework which posits guidelines for the iterative relationship between data collection and analysis [37, 38]. This approach enables researchers to inductively “discover” categories, themes, and patterns that emerge from the data [37], and is shown in Fig. 1.

In phase 1 of the analysis, five interviews were undertaken and two researchers (KA and SD) read and re-read 3 transcripts and gave meaningful segments of text descriptive codes. The researchers met together to identify areas of similarity and differences and agreed upon an initial coding framework. KA then coded the remaining transcripts using this coding framework. In Phase 2, KA carried out a further 14 interviews, which were coded on NVivo 12 (QSR International) to support data collation. The coding was reviewed regularly by KA and SD, who also undertook constant comparison techniques. This involved the comparison of new data and themes with previous coding and transcripts to further develop codes and begin to identify relationships between codes. The process was supported by memo writing, namely identifying areas of uncertainty and possible theoretical explanations, and at this stage four themes were perceived to need further exploration. This involved theoretical sampling, i.e. further data was collected with participants with specific features, in this case, we sought women, those with children, and older PLWH (over 70 years). In phase 3, further interviews with 8 participants took place, themes were further reviewed using constant comparison techniques with the wider research team involved, and seven final conceptual domains were agreed upon. These final domains were identified inductively from the analysis, rather than from existing QoL models, each including sub-themes which were grouped as being the most appropriate fit within each specific domain.

## Results

### Study participants

Twenty-five PLWH with CI completed interviews. This included 18 men and 7 women, with an age range of 38–80 years. See Table 2 for descriptive data on participant characteristics.

**Table 2:** Socio-demographic and clinical characteristics of participants (*n* = 25)

Characteristic	*n*
Number of female participants (%)	7 (28)
Median age in years (range)	56 (35–80)
Age (*n*, %)	
35–50	8 (32)
51–65	13 (52)
66–80	4 (16)
Ethnicity (*n*, %)	
White British	11 (44)
Black African	8 (32)
Black Caribbean	1 (4)
White—other	3 (12)
Black and white mixed race	2 (8)
Sexuality (*n*, %)	
MSM	14 (56)
Heterosexual	10 (40)
Other	1 (4)
Relationship status (*n*, %)	
Single	16 (64)
In a relationship/married/ civil partnership	9 (36)
Employment status (*n*, %)	
Not currently employed	15 (60)
Employed (full or part time)	6 (24)
Retired	4 (16)
Household income (*n*, %)	
Under £20,000	19 (76)
£20,000–£30,000	1 (4)
£30,000–£50,000	3 (12)
Over £50,000	1 (4)
Highest level of education (*n*, %)	
Secondary education/ GCSE’s	11 (44)
Sixth form/A or O-levels	3 (12)
Degree (Undergraduate/postgraduate)	9 (36)
Neurocognitive severity (MoCA score) (*n*, %)	
Mild CI (18–25)	11 (44)
Moderate CI (10–17)	10 (40)
Severe CI (< 10)	4 (16)
Median number of co-morbidities (range)	5 (0–17)
Median time since HIV diagnosis in years (range)	17 (1–34)
Median duration on cART in years (range)	14 (1–24)

*N.B. MSM* men who have sex with men

### Key domains

Using continued constant comparison techniques among the study team, first yielded six overarching domains which was then expanded to seven following whole study team consultation. The seven interrelated conceptual domains each had a number of descriptive components (Table 3). Each of the seven domains is described below from the perspective of PLWH with CI.

**Table 3:** Summary of domains (*n* = 7)

Domain	Examples
(1) Physical function: ability to perform normal activities of daily life Components: instrumental activities of daily, living recreational activities, employment/vocational activities	(1) I can’t use a cashpoint, because I kept taking the card out but forgetting and leaving my money, I think, or I can’t use a self-service in a supermarket…I have to queue with a trolley, because I get…I don’t…I can’t… do things anymore (P05: Male, 64, severe CI)
	(2) Quality of life I would think is (to) do things and get out…which I don’t do really anymore, I’ll be honest…I’d go up [to bed] at 3 o’clock, put the TV on, watch that with the dogs…that’s my choice, I don’t have to, but it’s just easier that way. (P02: Male, 55, moderate CI)
	(3) I thank god for work…I think work have been really good at supporting me mentally, my manager knows about my [cognitive] problems and they are quite flexi, I can take time out…I’m well supported, it makes a huge difference to everything (P23: Female, 49, mild CI)
(2) Cognition: Cognitive symptoms Components: memory and executive functioning, concentration/attention, communication	(4) Sometimes it gets very hard and bad because you are round and round on the same thing, you get frustrated and anxious about what you have done or what things you are supposed to do on that day. Especially, if I’m somewhere busy…its like your memory feels as though you’ve got a cloud, a fog over it that impacts everything (P15: Female, 63, moderate CI)
	(5) It’s a daily struggle to remember things and concentrate… if I’m having a bad day, with my brain…it’s like I can’t do anything…I will try to go to work, but I can’t even concentrate on what my boss is saying to me, or what my friends at work at saying…and it’s so annoying cause, upsetting actually, like, I don’t want to be this useless person (P07: Male, 64, moderate CI)
	(6) Yeah, it’s difficult isn’t it? Not being able to stand up and say what I want is really frustrating, and I don’t want to be “Who’s that idiot over there?…there is this sense I’ve lost it completely (P10: male, 68, Severe CI)
(3) Social connectedness: ability and willingness to form and maintain social relationship Components: social engagement and social withdrawal, social support	(7) You end up slowly getting rid of them [friends]…you start to put distance because…it’s like not wanting to meet people because it was difficult and I don’t want to go…you then start having missed calls and you think “Oh no, they want that, I can’t cope with it”. So…everybody starts to pick up [on it], like, “Oh he’s…” so I lost a lot of friends through that. (P05: Male, 64, severe CI)
	(8) I don’t have any friends, so I don’t see anybody…I’ve never had many friends and now I definitely don’t, I stopped when my memory happened…I’ll struggle or look stupid, and I can’t get there so it’s a no–no…I speak to the neighbour and my mother, but she’s in Scotland so I never see her, neither of us would travel (P18, Male, 49, mild CI)
	(9) I think the fact I don’t live on my own, I’ve got my daughter, she’s 17, my husband, and I speak to my sisters all the time…I’ve got friends…most of them are nurses…I’ve got really nice neighbours…I’ve got quite a few things which help a lot to be honest, they keep me going [laughs] (P23: Female, 47, mild CI)
(4) Stigma: experiences of stigma and discrimination and impact on self (related to both HIV and CI) Component: enacted and self-stigma, barriers to help-seeking (social, medical, welfare)	(10) Good QOL is being able to tell people what is wrong, have someone to talk to you know? (P17: Male, 59, mild CI)
	(11) Perhaps it does get in the way of you accessing other healthcare…I was resistant to go to the doctors again about my memory stuff, because of having to bring up the HIV stuff again…so that’s why sometimes people will not tell anybody there is a problem (P07: Male, 64, moderate CI)
	(12) It led back [when I spoke about cognitive problems to employers] …when she knew I was HIV, she told another woman, and they were so rude, awful to me. I left, [and] now I worry about working and it’s just affected me…how I feel about myself, it was very traumatic, very upsetting (P13: Female, 52, mild CI)
(5) Self- concept: perception of self Components: self- esteem and confidence, identity, sense of independence, concerns about the future (future-self), attitude and personality	(13) It’s just another thing, I’ve not achieved much because of my illness [HIV], and this is the last straw really…in terms of me doing anything productive or getting a job… there’s not much hope for me, you know, making much of myself (P11: Male, 38, mild CI)
	(14) You’re not yourself, you’re not yourself, it’s like you’ve lost yourself…when you can’t remember anything, constantly relying on people to remind you to do stuff. I think it’s horrible and it’s got a great impact on the quality of life you can have (P23: Female, 47, mild CI)
	(15) I’m a strong person, after the shock of it [CI diagnosis] but I’m resilient, I know this. You just have to try to enjoy life, not worry too much and get on with things (P25: Male, 55, mild CI)
	(16) I do worry about it [cognitive deterioration], but in a jocular manner…I worry about forgetting my family, them having to deal with me, my goodness, that worries me…it’s worrying to know that one person can have a disease which makes you forget who you are…but it happens. We can’t help it, can we? (P19: female, 61, mild CI)
(6) Acceptance and control: acceptance and understanding of health conditions and perceived control over health outcomes Components: understanding of health conditions, sense of control over health outcomes, Employment of compensatory and remediation strategies	(17) I was grieving about it, given what you are losing basically and then [I’ve] come to terms with it, with HIV, I can actually say that I have mastered it…because I’ve been actually trying to understand it in full, how it works…and there are actually lots of people with HIV so it doesn’t particularly scare me anymore, I take my pills and I should be okay…I am learning about my memory now and it won’t necessary get worse, which makes me feel better (P01: Male, 38, moderate CI)
	(18) It’s from what we call a demon, I find myself sick…I think continually like taking medication, I don’t know what it is, what it’s for…I don’t understand, and I think where has my life gone…maybe I’m not sure [what caused my CI], I’m hoping you can tell me, so then I can know what to do about it (P24: Female, 45, mild CI)
(7) Physical and mental health and wellbeing Components: physical health, mental health, global health appraisal and life satisfaction	(19) I do mindfulness and some yoga now, which I think makes me more present with my memory problems so I’m going to forget things less…well that’s the idea, and I am drinking a lot less now which I’ve been told will help…yes, there is some hope for me I think (P25: Male, 57, mild CI)
	(20) This has just thrown me a bit, I’ve been good the last 10–15 years [referring to prior depression], been able to get on and stuff…but it’s very, very worrying, forgetting things all time, it does, it does make me feel pretty miserable if I’m being honest (P06: Male, 50, mild CI)
	(21) I just like to look out there, I think of the friends I’ve lost and go “Wow, how lucky am I, you know, to be alive and to be able to experience this”, and I like to check in with myself every now and again…just to thank my lucky stars (No.09: Male, 44, mild CI)
	(22) It isn’t something that’s easy [CI]…It doesn’t make you a very happy person. And happiness depends on everything…on the state of your mind being able to cope with life, it’s just always there (P24: Female, 45, mild CI)

### Physical function

For PLWH with CI difficulties performing at least some instrumental activities of daily living (ADL) (e.g., cooking, shopping, food preparation, housekeeping, laundry, managing finances, managing medications, using technology) were reported by all to varying degrees, and appeared dependent on CI severity (component: activities of daily living). For everyone we spoke to this negatively impacted self-concept/esteem and sense of independence (Table 3, example 1). PLWH with CI described attenuated activity levels since the onset of cognitive difficulties and described how they missed doing things previously enjoyed (e.g., reading, holidaying, going to concerts, dancing, seeing friends) (Table 3, example 2; component: recreational activities). For two participants, substitution for another activity helped to maintain positive self-appraisal. For many, PLWH with CI engaging in outside activities was described as effortful and stressful, with individuals expressing a strong preference for staying at home (Table 3, example 2). For eight participants, positive perceptions came from maintaining a sense of normality, particularly if they were able to maintain employment via workplace adaption or changing roles (component: employment and vocational activities). Not working was a considerable concern for many PLWH with CI, causing significant financial burden and increasing feelings of uselessness. Individuals reported concerns speaking to their employers about their cognitive issues, fearing they would be labelled as incompetent or that they would need to reveal the cause of their CI (i.e., HIV) resulting in anxiety and stress. For those able to speak to employers, workplace adaptation and continued employment was a strong moderator of good QoL appraisal (Table 3, example 3).

### Cognition

PLWH with CI described issues with short-term recall which impacted a range of activities. Depending on CI severity, individuals described difficulty remembering past events, names or faces, and dates and times (including those of appointments or meetings). Difficulties multi-tasking impacted ADLs and many described their CI as a ‘mental fog’ which led to their feeling overwhelmed in busy or loud environments, and resulted in information processing difficulties (Table 3, example 4; component: memory and executive functioning). All PLWH with CI reported problems attending to and concentrating on activities, along with feelings of restlessness, which impacted their ability to engage in activities lowering self-efficacy and HRQoL (Table 3, example 5; component: concentration and attention). Particularly troubling for a number of participants was the impact their CI had on their ability to communicate, this again varied depending on CI severity. Many described word-finding difficulties, issues with expressing themselves, and trouble remembering conversations, which was described as embarrassing and frustrating (Table 3, example 6; component: communication).

### Social connectedness

In the majority of cases, a consequence of cognitive difficulties was social withdrawal. Many PLWH with CI described feeling overwhelmed, distracted, and stressed when in group situations or busy environments, preferring only to socialise one-on-one in quiet places or not at all. Social withdrawal was described as a protective strategy by four participants; one which was necessary, despite its negative impact on HRQoL. Participants described difficulties maintaining social networks (Table 3, example 7). However, where social networks could be maintained, these provided emotional support and consolidation of identity (component: social engagement and withdrawal). While everyone we spoke to described attenuation of social activities following onset of CI, for 18 participants this appeared to compound an already limited social life with few avenues for support: many participants (particularly homosexual males) described limited social contact, with no partner, children, few friends, and limited contact with families (Table 3, example 8). In contrast, for those participants who had strong support networks, higher levels of social engagement and lower levels of social withdrawal were reported. Having a supportive partner, children, and friends with which they could discuss health concerns openly appeared to be a considerable moderator of HRQoL, which, along with conferring positive affect and reassurance, provided functional assistance and hope (Table 3, example 9; component: social support). Seven PLWH with CI described their faith and the church environment as a source of emotional and social support which conferred broad quality of life benefits: allowing them to feel confident socialising, while specific rituals in their religious practice assisted with their day-to-day wellbeing, conferring hope and resilience. Despite this all of the participants who reported attending a church stated that they would not tell their church-based friends about their HIV status.

### Stigma

PLWH with CI described considerable variation in community or cultural attitudes towards HIV. Black heterosexual participants all described the stigma and discrimination they would face if others in their community became aware of their HIV status, which affected their ability to discuss cognitive concerns, and so consequently limited their receipt of social support (Table 3, example 10; component: barrier to helpseeking). Two individuals reported concerns regarding the care they could expect to receive considering their HIV status, should their CI worsen. Similarly, three PLWH with CI described how having HIV attenuates helpseeking for cognitive problems, with individuals reticent to seek medical or welfare benefits due to fears around having to reveal their HIV status (Table 3, example 11; component: barrier to helpseeking). PLWH with CI all recalled instances of discrimination, prejudice, or enmity due to their HIV status. Two individuals recalled recent instances of discrimination at work when seeking support for cognitive difficulties led to the disclosure of their HIV status (component: enacted stigma); this resulted in continued hesitancy regarding future employment, financial insecurity, reduced self-esteem (Table 3, example 12; component: self-stigma) and effected QoL.

### Self-concept

PLWH reflected on the how their CI had impacted negatively on their self-esteem, confidence, and independence, with many, referring to themselves using derogatory language, such as “the village idiot”, “useless”, “worthless” and “stupid” (component: self-esteem and confidence). Individuals who required additional support expressed ambivalence: appreciating the help provided by friends or family while expressing sadness and in some cases guilt at their perceived sense of burden (component: sense of independence). CI appeared to compound already low self-esteem experienced by many as a consequence of living with HIV and, in many cases, having concurrent mental health problems (Table 3, example 13). This impact on self-esteem, coupled with changes in functional abilities, independence, and social difficulties, led to a sense of identity loss for a number of PLWH with CI we spoke to (Table 3, example 14). Participants described being ‘quieter’ and ‘reserved’ particularly in social situations and spoke with some incredulity and loss when considering their former selves (component: identity). However, many were able to draw on their experiences of living with HIV, which led them to describe themselves as resilient individuals, able to cope with adversity, and who felt the difficulties they had experienced previously helped equip them to deal with their cognitive problems. They spoke of the importance of maintaining a positive attitude, accepting one’s difficulties and ‘getting on with it’ (Table 3, example 15; component: attitude and personality). This attitude helped combat the fears expressed by many regarding their cognitive health deteriorating, financial insecurities, and becoming a burden to friends and family (Table 3, example 16; component: future self).

### Control and acceptance

PLWH with CI who understood the basic pathology of HIV and could describe their cognitive issues tended to report better HRQoL (Table 3, example 17; component: understanding of health conditions). These individuals appeared to compartmentalise their health, seeing and treating health problems (including HIV and CI) as distinct issues. Those reporting worse HRQoL saw their health globally, which increased perceived burden of problems and made it harder to address specific difficulties. Two Black women we spoke to described their illnesses as the result of a ‘curse’—this spiritual attribution, hugely increased feeling of distress and powerlessness over health outcomes (Table 3, example 18; component: perceived control over health outcomes). Individuals reported positive experiences of attending HIV memory services; being listened to and assessed was described as reassuring and it allowed participants to begin to understand and address their cognitive difficulties. Understanding and acknowledging CI allowed PLWH to begin to implement strategies which, along with improving functioning, alleviated stress and anxiety, in turn improving HRQoL. Use of compensatory strategies (e.g., diaries, calendars, notes, alarms, along with support from family or friends) facilitated function, independence, and hopefulness regarding their health outcomes (component: compensatory strategies). Remediation strategies (e.g., yoga, mindfulness, general exercise, brain-training games, volunteering, lifestyle changes (e.g., reducing alcohol consumption) further enhanced a sense of control over health outcomes, along with decreasing concerns regarding cognitive deterioration in the future (Table 3, example 19; component: remediation strategies).

### Physical and psychological health and wellbeing

PLWH with CI tended to describe their overall health negatively reporting significant burdens of mental and physical co-morbidities (related and un-related to HIV) (component: mental health, physical health) along with CI which impacted on HRQoL (component: global health appraisal). Particularly striking was the high prevalence of mental health issues, which were reported to have been started or exacerbated by the onset of cognitive problems (Table 3, example 20; component: mental health). Interestingly, five participants framed their difficulties in the context of having survived the AIDS crisis and compared themselves to peers they had lost during this time, describing themselves as ‘one of the lucky ones’ (Table 3, example 21). Participants tended to refer to their health-related difficulties in terms of their ability to cope, and whether they were able to feel happiness or, failing that, contentment (Table 3, example 22). PLWH with CI reported struggling with the relatively uncertain trajectory of impairments, however, for those reporting better HRQoL, strategies which increased perceived control over health outcomes (e.g., lifestyle changes) appeared important. These individuals reported better perceptions of coping and expressed optimistic attitudes towards their cognitive difficulties and overall HRQoL.

## Discussion

This study represents the first qualitative study directly exploring HRQoL in PLWH with CI. It provides unique data from a small sample of PLWH with CI on how living with CI impacts and influences HRQoL in PLWH, identifying domains and descriptive components which make up HRQoL in this population. Seven interrelated domains were identified: Physical function, Cognition, Social connectedness, Physical and Mental health and wellbeing, Stigma, Self-concept and Control and Acceptance. Overall, the participants appeared to describe their HRQoL in negative terms. Many described their cognitive symptoms as impairing functional abilities, including recreational and social engagement. Individuals identified the detrimental effect that this had on their self-esteem and identity, and how living with CI in the context of HIV, was moderated by the impact of stigma on their lives and their perceived sense of control over health outcomes. Additionally, many described how living with CI effected their mental health, with participants describing high levels of depression and anxiety generally, and when considering the relatively uncertain trajectory of their CI.

Four domains identified in this study are consistent with findings from the wider literature, including attenuated function [39–43], and impairments in cognition [2, 28, 44] impacting on HRQoL appraisal. Physical and mental health and wellbeing is supported as a domain as PLWH with CI report worse summative physical [2, 30–32] and mental [30–32] HRQoL scores. HIV-stigma is a well reported barrier to conversations with friends, family and healthcare professionals about health [2, 45], and as in another study [2], participants provided examples of self or enacted stigma which prevented them from talking about their cognitive difficulties. Along with broader information campaigns on HIV-stigma and its impacts, interventions aimed at improving knowledge and facilitating communication of HIV-associated functional health difficulties among friends, family, employers, and healthcare professionals, might help reduce stigma and subsequently improve support [46].

This study contributes to three areas where literature is limited. First, we found a particular impact on social connectedness, with participants reporting high levels of social withdrawal which negatively affected HRQoL and limited receipt of social support. Within this, a novel finding is the role of religion or religious community on HRQoL. Individuals described the social support and safety felt within their religious communities yet reported how discussion of health concerns, specifically HIV or HIV-related illnesses, would not be appropriate or well received. This warrants further examination. We further identified the importance of self-concept on HRQoL in PLWH with CI, the subthemes of identity, attitudes and personality, and future self-concerns, are relatively unexplored. Self-concept has been identified as an important domain of HRQoL for individuals with dementia [23], but is not captured in HIV-specific conceptualisations. Finally, among our participants we found factors related to the control and acceptance of CI to be important moderators on HRQoL, which require further exploration. While other studies have reported on the use of compensatory or remediation strategies in PLWH with CI [2], we extend this to show how among our participants acknowledgement and understanding of conditions facilitates strategy implementation and increases perceived control over health-outcomes, which in turn improves HRQoL. Interventions, which, from the point of diagnosis forward, regularly review and remind patients of factors impacting cognitive health, along with collaborative practical assistance which engender confidence and empowerment, may be helpful in instilling a sense of control over health outcomes and improving overall QoL appraisal.

This study has important limitations, it was an exploratory study limited to urban south-east England. The sample was made up of predominately MSM and Black heterosexual women all of whom were accessing specialist HIV services, and as such is unlikely to be fully representative of HIV populations in the UK. Furthermore, there may be different experiences of HRQoL in PLWH with CI in mainstream services, not examined in the current study. Due to our limited sample we did not explore certain demographic and clinical differences (e.g. including duration of time on antiretroviral therapy, time since HIV diagnosis and other lifestyle factors) across our sample. Similarly, among our participants the social context within which one lives with HIV appeared to be a strong moderator of life quality in the context of CI and we noted cultural differences between MSM and Black heterosexual women which would benefit from further study as would other groups.

There are, however, three main strengths to the study. First, it is the first study to examine and delineate the domains impacting HRQoL in those with HIV and CI. The World Health Organization has identified quality of life as an essential consideration in the care of PLWH [47] and there have been recent calls to include it as a ‘fourth 90’ in the 90–90-90 testing and treatment targets proposed in 2016 [47, 48]. With an ageing HIV population, we know the numbers with CI will increase, policy in this area will need to change, and HIV and memory services will need to expand and adapt to the specific needs of this group. Second, using a good quality qualitative, ‘bottom up’ approach to data collection and analysis, we have enhanced the validity of results and accessed the experiences of this group to generate new insights into HRQoL in PLWH with CI. Third, the conceptualisation of HRQoL we have identified indicates there are domains of HRQoL which are not captured in generic, HIV, or dementia-specific conceptualisations. This appreciation of condition-specific multidimensionality helps clinical understanding of illness impact, and therefore can help to inform the development of tailored interventions to improve outcomes for PLWH with CI.

## Conclusions

This study offers new evidence to help understand the HRQoL of PLWH with CI. We provide evidence suggestive of the poor HRQoL experienced by many PLWH with CI and identify domains where future care, policy and research developments could be focused. Caution is, however, encouraged given that this was a small sample comprised of predominately MSM and Black heterosexual women in the South-East of England. Future research should examine the relationships between domains identified and how different sociodemographic and clinical factors influence HRQoL. Moreover, the experiences of other key groups, such as Black heterosexual men and transgender PLWH should be considered in qualitative work going forward.
